# Experimental demonstration of confidential communication with quantum security monitoring

**DOI:** 10.1038/s41598-021-01013-y

**Published:** 2021-11-04

**Authors:** Yupeng Gong, Adrian Wonfor, Jeffrey H. Hunt, Ian H. White, Richard V. Penty

**Affiliations:** 1 Electrical Engineering Division, University of Cambridge, 9 JJ Thomson Ave, Cambridge, CB3 0FA UK; 2 The Boeing Company, Chicago, IL USA; 3 University of Bath, Claverton Down, Bath, BA2 7AY UK

**Keywords:** Fibre optics and optical communications, Quantum optics

## Abstract

Security issues and attack management of optical communication have come increasingly important. Quantum techniques are explored to secure or protect classical communication. In this paper, we present a method for in-service optical physical layer security monitoring that has vacuum-noise level sensitivity without classical security loopholes. This quantum-based method of eavesdropping detection, similar to that used in conventional pilot tone systems, is achieved by sending quantum signals, here comprised of continuous variable quantum states, i.e. weak coherent states modulated at the quantum level. An experimental demonstration of attack detection using the technique was presented for an ideal fibre tapping attack that taps 1% of the ongoing light in a 10 dB channel, and also an ideal correlated jamming attack in the same channel that maintains the light power with excess noise increased by 0.5 shot noise unit. The quantum monitoring system monitors suspicious changes in the quantum signal with the help of advanced data processing algorithms. In addition, unlike the CV-QKD system which is very sensitive to channel excess noise and receiver system noise, the quantum monitoring is potentially more compatible with current optical infrastructure, as it lowers the system requirements and potentially allows much higher classical data rate communication with links length up to 100 s km.

## Introduction

In the era of “big data”, optical fiber networks are growing to accommodate massive, anticipated capacity demands^1,2^. At the same time, applications using confidential information, e.g. for the transmission of financial, medical and national security data, increases the necessity for data to be transmitted securely^3^. In addition to optical encryption methods^4,5^, classical physical layer security protection relies on active fiber monitoring techniques. These are generally based on statistical analysis of the received signal, e.g. by measuring the mean optical power^6^, or incorporating active diagnostics via sending a separate signal into the network^7,8^, e.g. using optical time domain reflectometry (OTDR)^9,10^. In practice, such classical methods have their own vulnerabilities and security loopholes^11,12^. They can, for example, be compromised by an optical intercept-resend attack which intercepts the signal and resends an identical replica after capturing the data^13,14^, or by a correlated jamming attack which maintains the link optical power after tapping by replacing the tapped light with noise. In addition, a sophisticated fiber tapping attack could perturb the fiber transmission by less than 0.1 dB^12^, making real-time monitoring of the link power at the required (high) precision both challenging and costly. A detailed introduction to various current active fiber monitoring techniques and their security loopholes can be found in Refs.^11,15,16^.

Additional vulnerabilities are on the horizon, as the development of quantum computers may break current cryptography systems that are based on computational complexity, such as factorization (RSA), discrete logarithms (Diffie–Hellman), and elliptic curves. Possible candidates to respond to this threat are post quantum (or quantum resistant) cryptography (PQC)^17^ which is a refinement of current cryptography not susceptible to Shor’s algorithm^18^, and quantum key distribution (QKD)^19^ which transmits secure keys using quantum states for classical symmetry encryption. Although the cost of integrating PQC into current cryptography systems is much less than QKD, it is still in a development stage with different approaches being explored, and as yet has no defined or agreed mechanism for implementation.

QKD requires legitimate users to encode random information, transmit, and measure quantum states. Its security derives from the quantum no-cloning theorem and can distill secure random keys using classical techniques, i.e. error-correction^20,21^ and privacy amplification^22,23^. Legitimate users can provide an upper bound of leaked information with an arbitrarily small quantity of information available to an eavesdropper. Substantial development has taken place in both the theoretical and experimental aspects of QKD, with various variants being proposed, e.g. protocols based on continuous variable (CV)^24,25^ and discrete variable (DV) quantum states^26,27^, and also Differential phase reference protocols^28,29^. Recently, protocols with reduced security constraints on devices, e.g. the measurement-device-independent (MDI)^30,31^ and device-independent (DI)^32^ protocols, have been developed. Experimental laboratory transmission over more than 400 km has been demonstrated using the twin-field protocol^33,34^ which exceeds the previous theoretical limit, i.e. the PLOB (Pirandola, Laurenza, Ottaviani & Banchi) bound on secure distance^35^. However, the use of QKD in commercial optical networks has been limited by practical challenges^36,37^. These include the extremely low secure key rate at long distances, the relatively high system cost, and the security vulnerabilities still present in practical systems^32^. QKD is an emerging technology with a few commercial implementations, but still far from widespread deployment.

Nonetheless, techniques arising from QKD development, that is encoding information onto quantum states, security checking by measuring the quantum signal parameters, and the application of the no-cloning theorem, have inspired many other techniques for secure communication based on quantum physics. For example, in quantum secure direct communication (QSDC)^38,39^, quantum states are used to transmit messages or deterministic keys directly. Analogously to QKD, the secure distance and communication rate is still far from satisfactory. This is because both techniques aim to achieve unconditional security, which considers any non-zero quantum bit error rate (QBER) in DV systems or excess noise in CV systems to be caused by the eavesdropper. This sets stringent requirements on the practical channel and other devices to keep the QBER or the excess noise below a predetermined low level. Also, in Ref.^40^, modulated continuous variable quantum states are employed for security sharing within a network.

However, unconditional security is not always necessary for the deployment of practical applications, and any type of real-world secure communication allows for compromise between high data rate and high security. For example, in Ref.^41^ quantum states are used for the quantum low probability of intercept (QLPI) protocol, while phase shift keying is used for transmission of the classical information over the quantum channel. Quantum data locking^42^ is employed to transmit messages at a higher rate. In addition, although an eavesdropper is considered only limited by the law of physics in the QKD security analysis, near term technologies will only enable an adversary to attack the physical layer via classical means, e.g. by tapping the fiber, which cannot avoid influencing the transmission. Hence, in Ref.^14^ entangled photon pairs are sent via a reference channel to monitor the link security via performing a Bell test^43^ at the receiver. A theoretical method of confidential communication with messages encoded on the continuous variable quantum states directly is proposed in Ref.^44^, with part of the transmitted states being used to monitor the security of an ideal channel. Operations would presume that legitimate users would halt the communication when the quantum excess noise exceeds a certain threshold. However, the communication efficiency is still restricted due to the quantum detection system and interference from the intrinsic noise of a practical channel.

With the intended goal of addressing these and other pragmatic concerns, we discuss repurposing the CVQKD system and report the successful demonstration of a novel technique of monitoring the security of high data-rate classical communication using the quantum states^45,46^. In this protocol, classical communication signals are embedded with quantum modulated monitoring signals, enabling simultaneous transmission. Measurements are made to ensure the potential eavesdropper cannot distinguish a quantum modulated and a classical communication signal. This technique focuses on monitoring the physical layer security of the channel and analyzing the quantum signal to identify both potential passive and active attacks. It also allows equipment similar to that in classical coherent communications systems to be used, so that implementation is expected to be low cost. We first introduce the protocol, followed by the attack detection performance simulation in the “Result section”. The expeirmental demonstration results are then presented with details included in the “Methods” section and a disscussion is given in the last section.

## Results

### The quantum monitoring protocol

**Figure 1:**
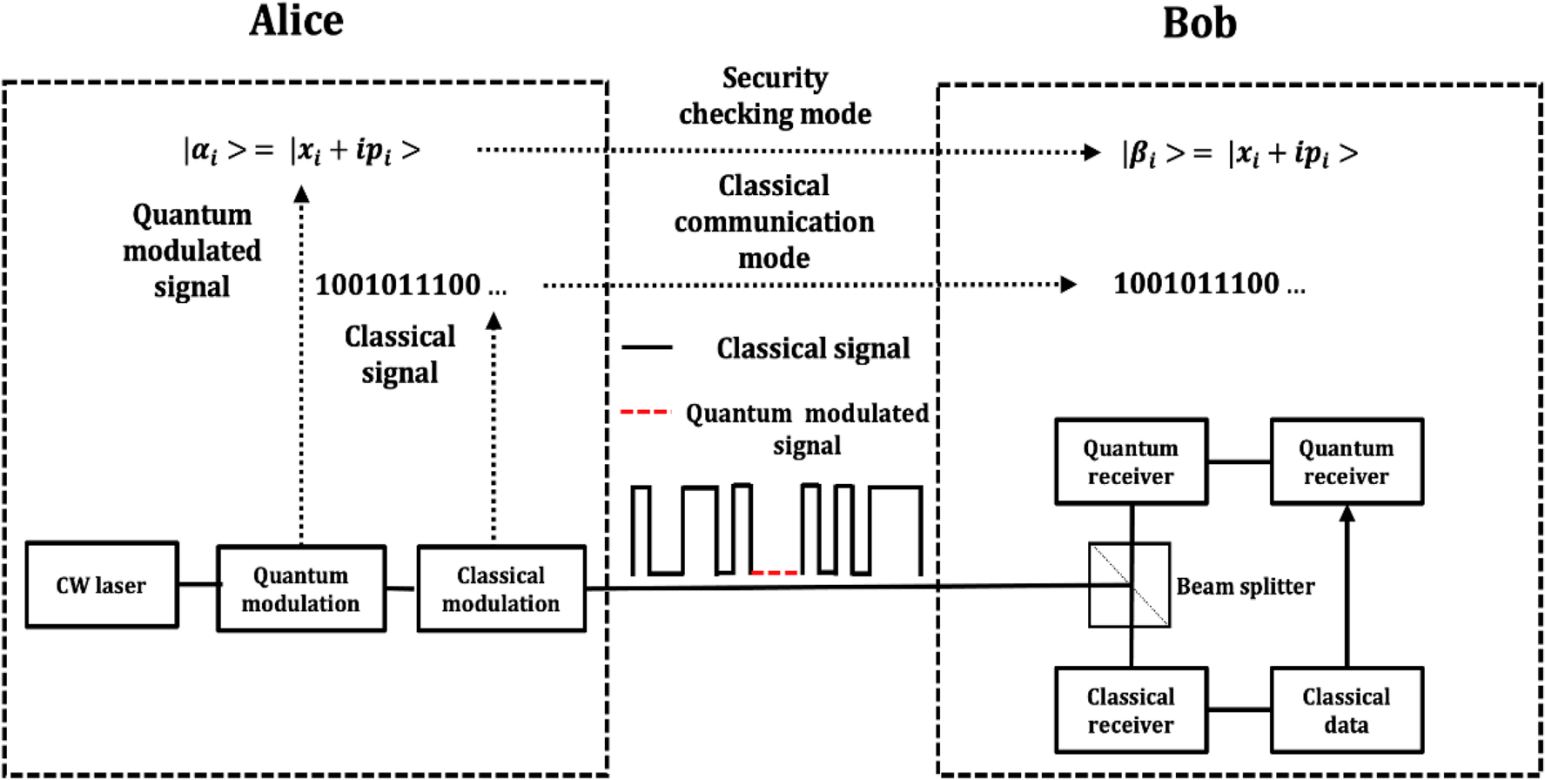
Block diagram of an example quantum alarm system. The transmitter consists of both classical and quantum modulation modules. Alice randomly chooses sending quantum modulated pilot tone signal and classical communication signal. At Bob’s side, both the quantum and classical signals are measured by the quantum and classical receivers respectively.

Our quantum monitoring system is based on the protocol proposed in Ref.^45^ using continuous variable quantum states. As shown in Fig. 1, the system has two operational modes, the security monitoring mode, and the classical communication mode. For the signal waveform, the black solid line represents the classical signal using amplitude shift keying (ASK) modulation schemes. The red dashed line represents the displaced quantum modulated signal whose length is less than that of maximum sequence of zeros permitted in the classical modulation scheme. The quantum transmitter consists of an amplitude and phase modulator while the quantum receiver is a low bandwidth homodyne/heterodyne detector. The sender, Alice, randomly switches between the two modes to insert quantum monitored signals into the classical communication signal. A splitter is used at the receiver, so that both the quantum and classical receivers measure the incoming signal. And the quantum receiver only keeps the information encoded in the security checking mode, where the quantum signal is measured to ensure link security. The classical signal encodes both classical communication information and the data required for the postprocessing of security checking.

Here, we introduce the four steps of the protocol.

1. Alice prepares the quantum modulated signal using CVQKD techniques, e.g. Gaussian modulation, binary modulation, four state modulation etc. Alice then displaces the signal to increase its intensity to the level of zeros in the classical ASK modulated signal.
2. Alice randomly switches between the classical ASK signal and the displaced quantum modulated signal.
3. Bob measures the signal and compares the information to Alice’s and calculates the quantum channel transmittance and excess noise.
4. Alice and Bob choose to halt or restore the communication if the error rate exceeds a certain threshold and trigger the alarm using appropriate data processing algorithms.

The crucial point is to make the quantum and classical signals indistinguishable to an eavesdropper so that she cannot avoid measuring the quantum-modulated signal when measuring the classical data. This can be achieved by transmitting the two modes simultaneously as proposed in Refs.^47,48^, where displacement is employed to transmit the CVQKD and classical coherent communication signals simultaneously. However, the relatively small bandwidth and measurement range of the quantum detector limits the practical application of this technique. Hence, we present an alternative technique that is able to make the quantum modulated signal indistinguishable from the classical communication signal. Specifically, the transmitter switches between quantum and classical modulated signal randomly using optical time division multiplexing (OTDM). Employing this, one can transmit them at the same wavelength over the same channel. The goal is to deceive the eavesdropper and make the quantum modulated signal equivalent to the classical modulated zeros. However, due to the small intensity of the quantum modulated signal, it needs to be amplitude displaced in the phase space to increase its intensity to the classical level of zeros. One can further increase the indistinguishability by including additional short bursts of zeros during the classical communications, akin to the decoy states used in QKD systems, or by replacing all classical zeros by the quantum modulated signal. The quantum modulated signal can be displaced to further increase the complexity of the receiver system. An analysis of the quantization noise for measuring quantum signal with extra displacement can be found in “Methods” section.

### Attack detection using quantum monitoring

**Figure 2:**
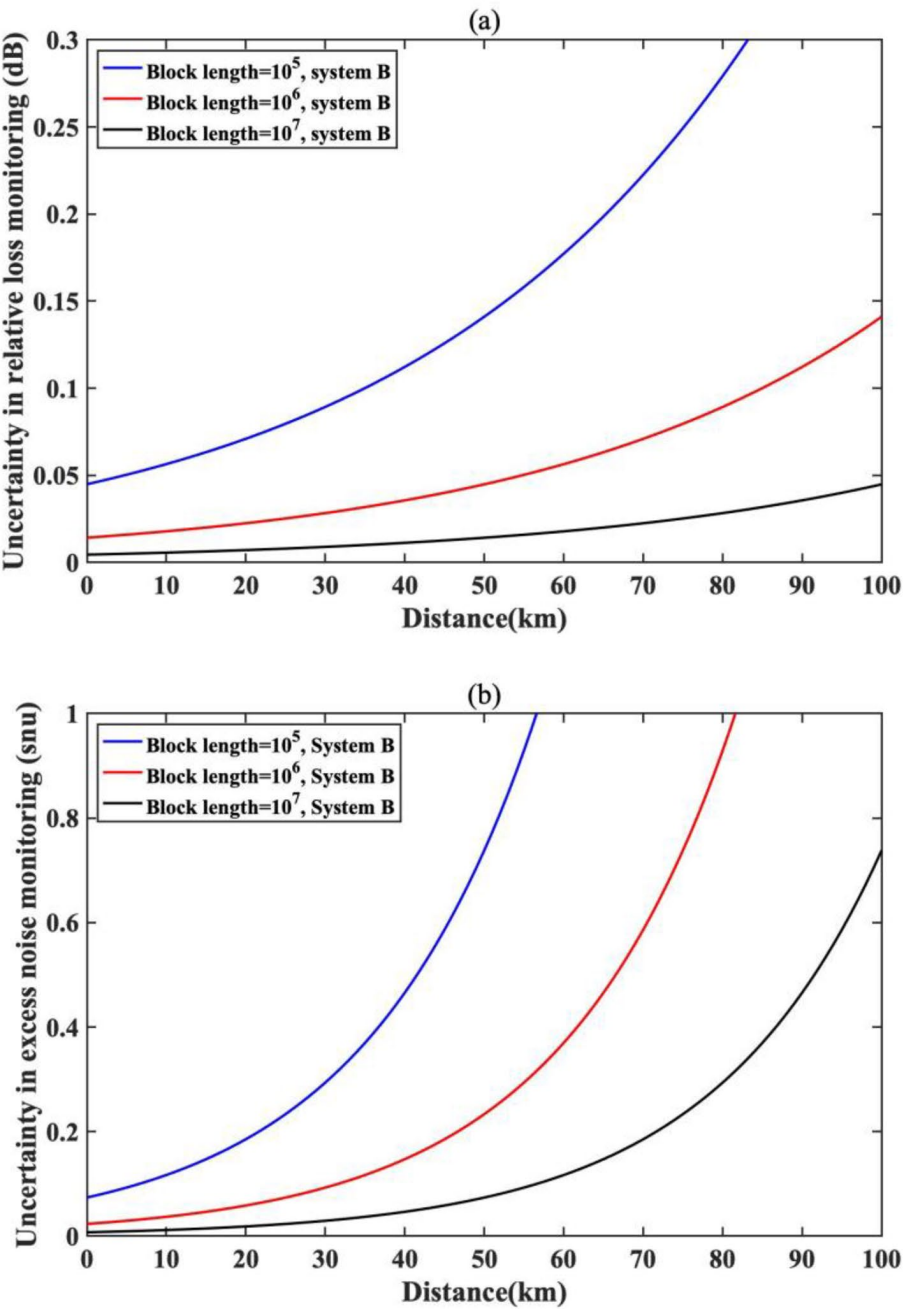
Accuracy of the quantum monitoring system. (**a**) Estimation uncertainty in loss monitoring. (**b**) Estimation uncertainty in excess noise monitoring.

The monitoring system characterizes the channel physical layer security condition by measuring the received quantum states. The estimation accuracy can be derived for both quantum channel loss $T$ and excess noise $\xi$ as follows based on the finite-size analysis in Ref.^49^:

$$T\sim \left[ {{{\left( {\hat{t} - Z_{{\frac{\varepsilon PE}{2}}} \sqrt {\frac{{\sigma^{2} }}{{mV_{A} }}} } \right)^{2} } \mathord{\left/ {\vphantom {{\left( {\hat{t} - Z_{{\frac{\varepsilon PE}{2}}} \sqrt {\frac{{\sigma^{2} }}{{mV_{A} }}} } \right)^{2} } \eta }} \right. \kern-\nulldelimiterspace} \eta },\frac{{\hat{t} + Z_{{\frac{\varepsilon PE}{2}}} \sqrt {\left. {\frac{{\sigma^{2} }}{{mV_{A} }}} \right)}^{2} }}{\eta }} \right]$$

$$\xi \sim \left[\widehat{\xi }-{Z}_{\frac{\epsilon PE}{2}}\frac{{\sigma }^{2}\sqrt{2}}{T\eta \sqrt{m}},\widehat{\xi }+{Z}_{\frac{\epsilon PE}{2}}\frac{{\sigma }^{2}\sqrt{2}}{T\eta \sqrt{m}}\right]$$

where ${V}_{A}$ is the modulation variance of the quantum signal,$\eta$ is the detector efficiency, $m$ is the monitoring block length, ${Z}_{\frac{\epsilon PE}{2}}$ is the confidence level. And ${\sigma }^{2}$ is the unknown noise variance and is given by $$\begin{array}{c}{\sigma }^{2}=1+\eta T \xi +{V}_{ele}\end{array}$$, where ${V}_{ele}$ is the detector electronic noise. The noise variance is normalized to the pre calibrated system shot noise unit (snu). We plot the accuracy of the technique in Fig. 2 at different distance with different monitoring block size.

As can be seen from the figure, we can adjust the accuracy by varying the monitoring data block length, the quantum modulation variance and the confidence level of the estimation for different monitoring applications. One can achieve much better accuracy than classical means without the need for the long block lengths that are required for QKD post-processing. In terms of quantum channel loss monitoring, we can achieve an accuracy of better than 0.1 dB over 100 km using a 10^7^ block length with 5 sigma (10^–7^) uncertainty. Thanks to the quantum detection system, we can achieve a quantum level accuracy of better than 1 system shot noise (snu) at 100 km, which is much more sensitive and accurate than classical techniques. The receiver system parameters are taken from^50^, where the quantum detector has electronic noise of 0.015 snu and a detector efficiency of 0.552.

In the case of attack detection using quantum monitoring, the ideal condition occurs when Alice and Bob are communicating through a lossless and noiseless channel, so that any increase in excess noise or loss is due to an eavesdropper. In practice, given the channel intrinsic noise and loss fluctuations, a good quantum monitoring protocol should enable Alice and Bob to communicate the entire message when there is no eavesdropper, i.e. avoid a false alarm, and have a quick response so that only a small amount of information is lost when there is an eavesdropper. Differentiation of a false alarm from an actual eavesdropping event can be achieved via exploring various statistical techniques, such as change point detection^51^, Bayesian change point detection^52^, the supervised learning algorithm^53^ and CUSUM (cumulative sum)^54^. In this paper, we employ the CUSUM algorithm which is able to identify the small changes within a large data set. Of course, one can employ a relative short block length with a faster reaction at the price of larger estimation uncertainty. A description of the CUSUM algorithm can be found in “Methods” section.

### Experimental demonstration of detecting physical layer attacks

As introduced in “Introduction”, the current physical layer attacks targeting the optical fiber systems can be broadly categorized as fiber tapping attacks and also signal jamming attacks. In this section, we present the experimental results of a proof of principle demonstration system under a simulated fiber tapping attack and a correlated jamming attack using a quantum alarm signal.

**Figure 3:**
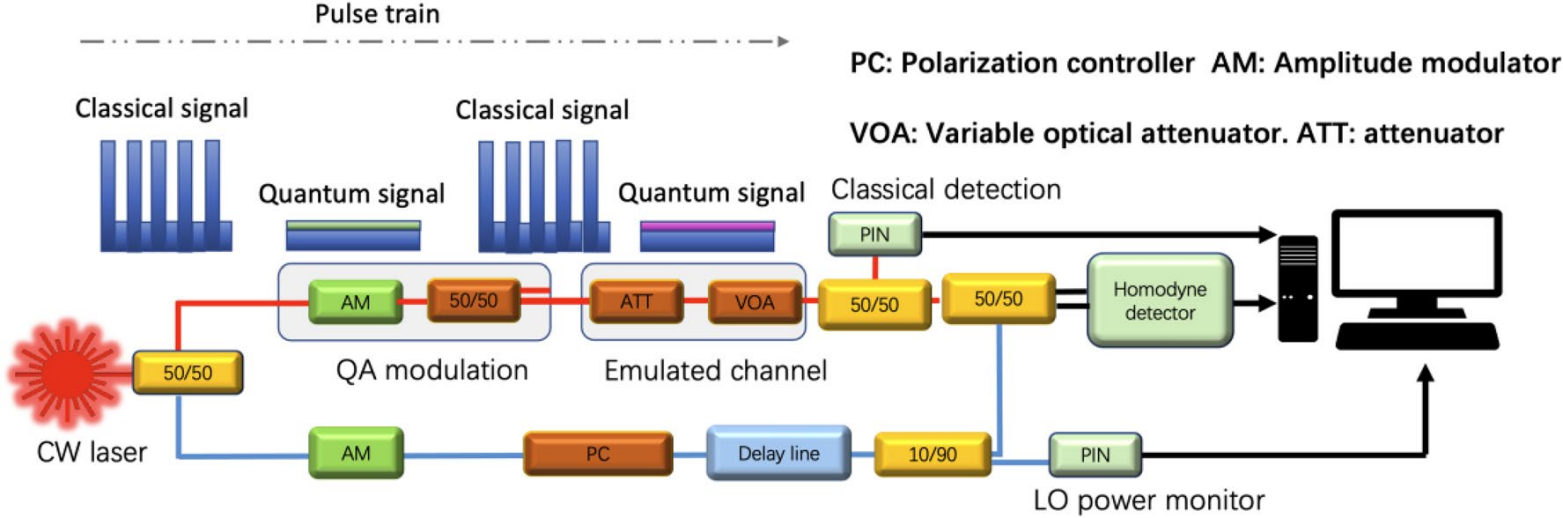
Experimental set-up of the proof-of-principle system. *ATT* attenuator, *VOA* variable optical attenuator, *AM* amplitude modulator, *PC* polarization controller, *PIN* photodiode, 50/50: 50:50 fibre coupler.

A diagram of the experimental set up is shown in Fig. 3 where we send the quantum modulated signal and classical signal simultaneously over the same channel. As described in the previous section, we randomly switch between sending classical modulated signals and quantum modulated signals using time division multiplexing (TDM) over the same channel with the same timeslot durations. For the classical signals, we encode random data using an on–off keying (OOK) modulation scheme. For the quantum signals, we employ a two-state modulation scheme^5^ that randomly sends one of two quantum states with the signal amplitude displaced to match the classical zero level. Hence, an eavesdropper cannot distinguish the quantum signals from the short bursts of classical zeros.

For the signal preparation (the red path in Fig. 3), we employ a single amplitude modulator to modulate both quantum and classical signals. A variable optical attenuator is used to emulate the optical channel with adjustable loss. At the receiver, a 50/50 splitter is used to direct half of the received signal for classical signal detection, with the other half transmitted to the quantum receiver, which comprises two balanced homodyne detectors to measure both x and p quadratures of the signals. The measured quantum and classical signals are both recorded for post-processing. In addition, for our initial demonstration, half of the power from the CW laser at the transmitter is pulsed and then connected to the quantum receiver as a local oscillator for homodyne measurement (the blue path in Fig. 3). In practical applications, this will be achieved by using a separate laser with the same wavelength at the receiver, similar to the approach used in classical optical coherent communication systems and the local local oscillator (LLO) CVQKD system proposed in Ref.^55^. In our tests, the system slot repetition rate is set as 25 MHz, with the classical data rate set at 1 Gbps. Physical layers attacks can be emulated by manipulating the emulated channel whose initial loss is set at 10 dB as the safe condition. In addition, we performed the quantum monitoring with a block length of 10^5^. Owing to the existence of classical signal and displacement, the average received optical power is − 30 dBm which much larger than conventional quantum communication system.

### Performance against a fiber tapping attack

Fiber tapping is a common type of physical layer attack utilizing the transparency of the optical fiber. During communication, light which propagates through the fiber guided in its core, keeping radiation from the fiber at a negligible level. However, an attacker targeting the physical layer can easily gain access to the light propagating in the core by intrusive or non-intrusive methods. Losses in the signal power caused by some of the tapping techniques may be less than 0.1 dB, which is barely detectable by conventional detection systems^2^. This kind of attack, especially aimed at industrial espionage, has a long history and known occurrences are reported in Ref.^6^. As a representative kind of passive attack, its detection is regarded as very difficult, since it does not alter the classical communication data. At the moment, the fiber network users do not have many options for securing data confidentiality and integrity against this attack. However, we will show that the quantum modulated signal is very sensitive to the classical fiber tapping attack and it can be detected effectively.

Specifically, in our proof of principle demonstration system as shown in Fig. 3, we realize the attack by pausing the real-time post-processing system and then inserting an additional splitter before the variable optical attenuator. This can be seen as the ideal case of a fiber tapping attack. That is, the attack happens immediately after the transmitter with very low insertion loss and no dramatic changes caused by bending or cutting the fiber. We then test the performance of the demonstration system under a 1% fiber tapping attack for a 10 dB overall loss fiber. This is equivalent to adding 0.04 dB loss to the original fiber and the transmission after attack is thus 0.099. We monitor and record both the signal and LO fluctuations so that they can be accounted for during post-processing. We collect the quantum measurement data for a total of 1800 samples. In addition, during points 1000–1500, we add the extra 1/99 splitter into the channel, so that the total loss is increased by 1%.

**Figure 4:**
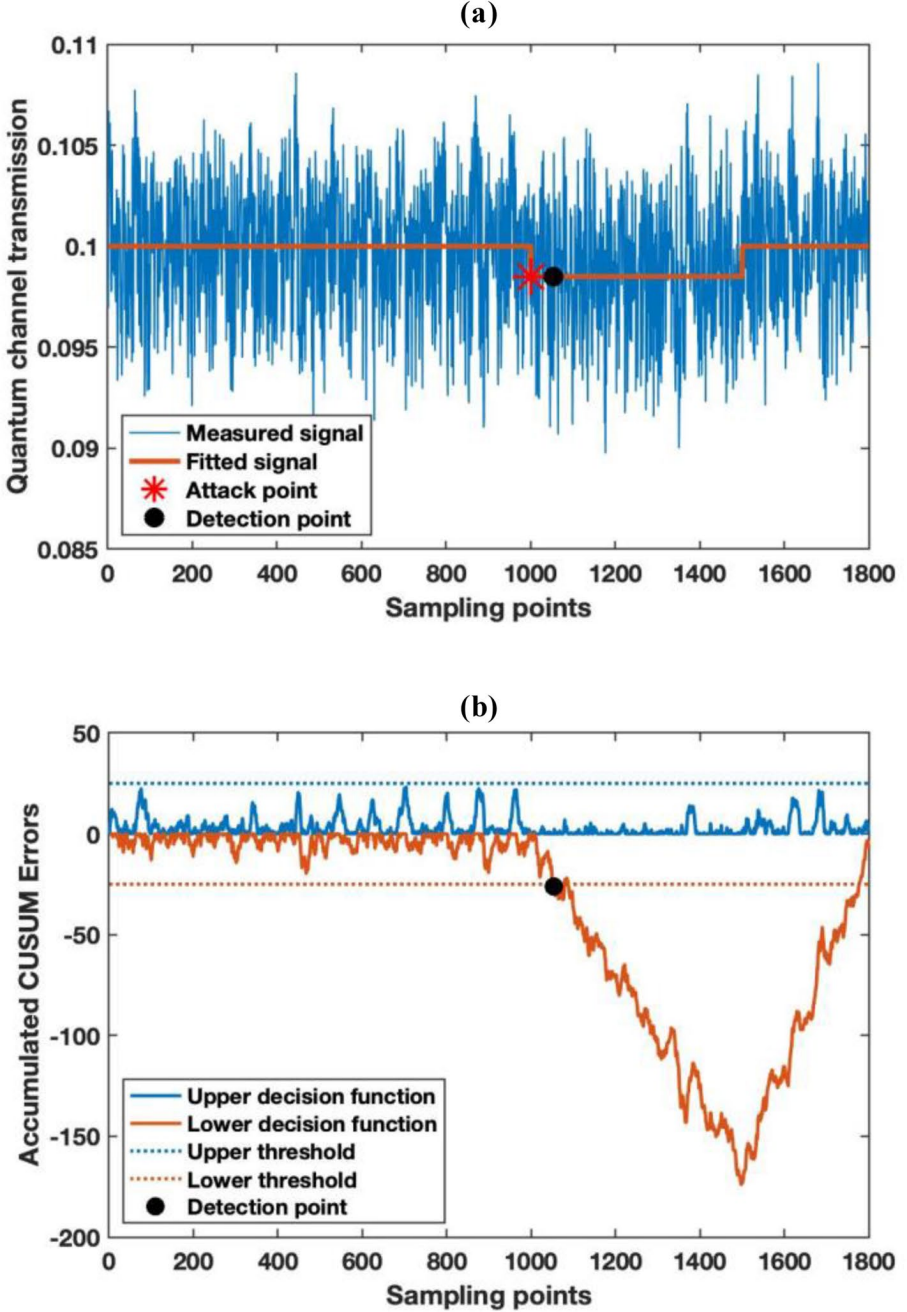
Monitoring result of channel loss under a fiber tapping attack and CUSUM analysis result. (**a**) The monitoring result of the quantum channel loss. (**b**) The accumulated CUSUM algorithm error for abrupt channel loss change detection.

The results of the monitoring performance are illustrated in Fig. 4. As can be seen, Fig. 4a illustrates the quantum channel loss monitoring results where the star represents the onset of the attack, and the circle is when the alarm is triggered. Owing to the estimation uncertainty calculated in “Discussion”, the change in channel loss cannot be identified immediately. However, with the help of CUSUM, we are able to plot the detailed accumulated errors of the decision function in Fig. 4b. The minimum detectable threshold of the algorithm is set at one unit of the standard deviation to reduce the incidence of false alarms. The upper and lower thresholds of the accumulated CUSUM errors are set at 25, which gives us a confidence level of more than 99.99%. In practice, the thresholds can be set at different levels regarding to the practical situation. The attack is initiated at sampling point 1000 and the alarm is triggered at sampling point 1053 with the lower decision function crosses the threshold, and so it only takes only 53 samples (0.424 s for 25 MHz repetition rate) for the attack to be detected. In addition, as illustrated in the figure, although the drift is very small (1%) and would take many more samples by detecting changes in the moving average, the errors are accumulated and caused a continuous decrease in the lower decision function of CUSUM result. The accumulated errors increase considerably and start to decrease when the attack terminates at sampling point 1500. We can also estimate the attack start and end points from the cumulative sum function after the decision function triggers the alarm.

### Performance against correlated jamming attack

We next test the performance in the presence of a correlated jamming attack. As introduced in “Introduction”, the correlated jamming attack is a form of new kind attack that taps a certain amount of signal while maintaining the channel optical power. It is able to deceive the classical attack detection techniques that are based simply on monitoring the optical power.

**Figure 5:**
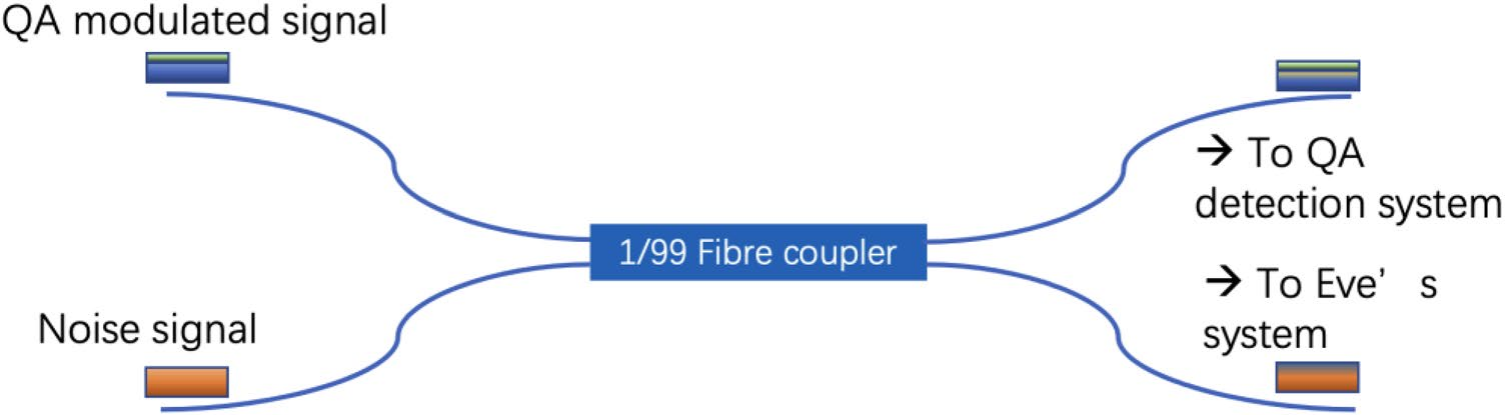
Implementation of the correlated jamming attack via an optical fibre coupler.

As can be seen in Fig. 5, to implement the correlated jamming attack, we again utilize a fibre coupler before the emulated channel. However, unlike in the fibre tapping attack case, measures are taken to maintain the optical power being injected into the channel. As a result, we split 1% of the QA signal and combine the rest with a noise signal from a similar laser. We adjust the injected laser power so that the optical powers before and after the attack are the same, so that the overall optical power being injected to the QA receiver is unaffected. Since the QA signal is at wavelength 1550 nm, the noise signal is an out-of-band jamming attack. This can be seen as an ideal correlated jamming attack, as the eavesdropper is able to split the ongoing light without interruption and only needs 1% of the light to decode the message.

**Figure 6:**
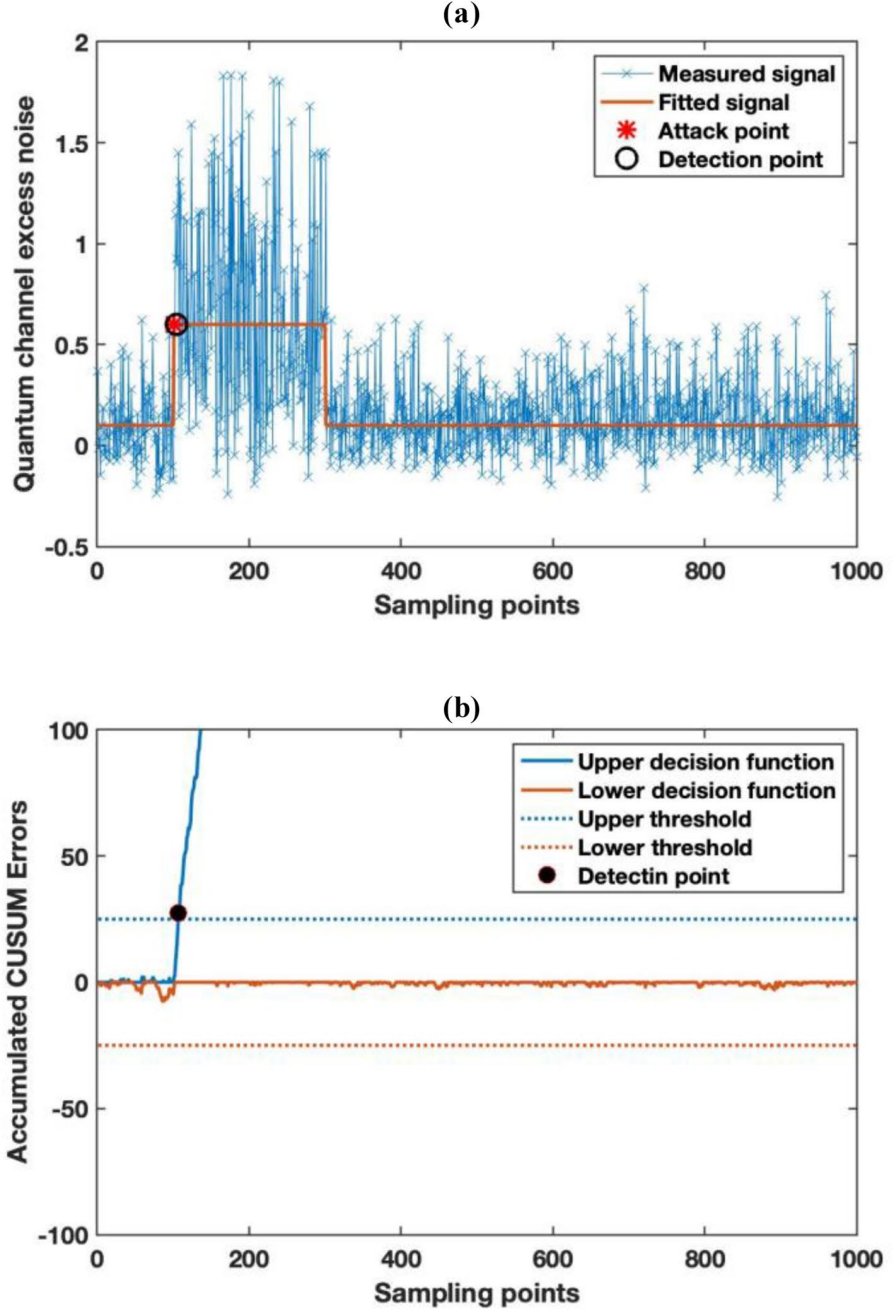
Monitoring result of quantum excess noise and CUSUM analysis result. (**a**) The monitoring result of the quantum channel excess noise. (**b**) The accumulated CUSUM algorithm error for abrupt channel excess noise change detection.

**Figure 7:**
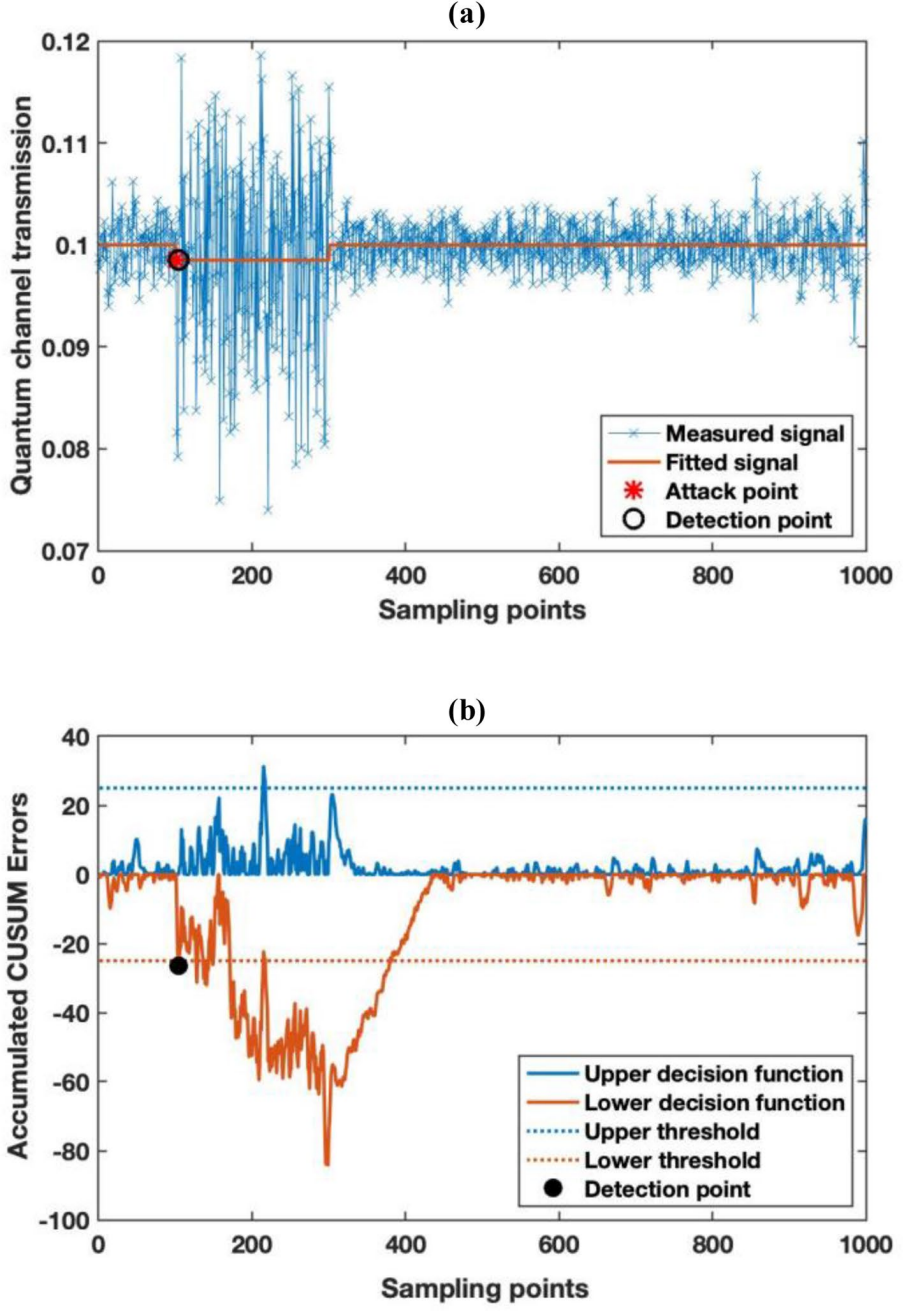
Monitoring result of quantum channel loss and CUSUM analysis result. (**a**) The monitoring result of the quantum channel loss. (**b**) The accumulated CUSUM algorithm error for abrupt channel loss change detection.

We then repeat the measurement and post-processing as for the fibre tapping attack. The monitoring results of quantum channel excess noise and loss are illustrated in Figs. 6, and 7, respectively. As can be seen from Fig. 6a, the mean quantum excess noise increases from 0.14 shot noise to 0.64 snu during the correlated jamming attack. Since, the baseline optical noise is one unit of shot noise, we can estimate the OSNR of the QA signal is decreased by 1 dB. From the empirical calculation in, we can estimate this level of reduction in OSNR would cause a trivial BER change in the classical signal. In practice, owing to the sporadic nature of this attack, this extra noise and temporal BER drop is very hard to detect with classical systems. Nonetheless, the increase it causes to the quantum excess noise is detectable, as it increases by almost a factor of 5 compared to its original value. We also applied the similar CUSUM analysis to the result. We set the alarm threshold by setting the minimum detectable mean shift of 1 $\sigma$ (0.2 snu) in the detected noise of the QA to avoid a false alarm. The alarm is triggered when CUSUM decision function crosses 25 accumulated errors, which is at sample no 105 in Fig. 6b. As a result, it only takes five samples (less than 0.04 s) of monitoring for the correlated jamming attack to be detected.

In addition, as illustrated in Fig. 7a, although the mean optical power is maintained, the measured quantum channel loss is still increased as the injected light is incoherent with the quantum signal. This is because the QA uses a coherent detector, the light injected in the attack to maintain optical power is incoherent with the LO at Bob and so the extra noise only influences the measurement fluctuation. Even with the injected light at the same wavelength as the signal, it is easy for the system to detect as it introduces additional noise. Moreover, the injected light is not identical to the quantum modulation, hence the quantum channel transmission will still be decreased. As shown in Fig. 7a similar to the 1% fiber tapping attack, we can clearly see a drop in the fitted signal result during the attack. However, the result has much larger fluctuation under attack compared to the standard fiber tapping attack. The measured standard deviation during the attack is about 0.009, i.e. 0.4 dB, while that during a fibre tapping attack is less than 0.0015, i.e. 0.04 dB. In addition, as shown in Fig. 7b, the alarm is triggered by the lower decision function at sample no 104, which takes many fewer samples than the fiber tapping attack, with reaction time of 0.032 s. Owing to the large fluctuation, the alarm is also triggered at the sample no 214 by the upper decision function. Hence, by considering both the transmission monitoring and excess noise monitoring results, we can conclude that a correlated jamming attack has taken place. In addition, the system is more sensitive to jamming attacks than to tapping attacks while most classical detection systems are very insensitive to jamming attacks.

## Discussion

QKD was proposed in response to the vulnerabilities of conventional cryptography in the face of future technology, i.e. the quantum computer^56^. However, the computational and communication complexity required for QKD, and hence its high cost, still restricts its use in large-scale optical communication networks. In addition, current eavesdroppers still rely on classical attacking methods that unavoidably introduce noise and a considerable level of extra loss. Hence, quantum monitoring provides another option for protecting information secrecy by ensuring the physical layer security and reforming the current active fiber monitoring technique.

In conclusion, we have proposed and demonstrated a quantum-based method of eavesdropping detection, similar to that used in conventional pilot tone systems, to monitor link security. This is achieved by sending quantum signals, here comprised of continuous variable quantum states, i.e. weak coherent states modulated at the quantum level. They are sensitive to measurement in the channel so that, as any unauthorized measurement introduces extra noise, which can be monitored to detect eavesdropping. Both the quantum signal excess noise and channel loss can be precisely estimated and monitored by real-time processing of the quantum signals. This process is similar to the parameter estimation step in continuous variable (CV) QKD systems, which however require use of more than half the quantum states to characterize the link security and estimate the secure key rate. Conversely, in this quantum monitoring system, all the quantum states are employed for security monitoring.

As analyzed in “Results”, the advantages of using quantum monitoring arise from the quantum nature of our security-checking signal and the sensitivity of quantum measurement. Specifically, compared to classical active fiber monitoring techniques, we can achieve much higher sensitivity to channel transmission changes (± 0.03 dB at 200 km) and also channel excess noise (± 1 snu at 200 km). We have demonstrated the ability to detect sporadic attacks with very fast detection. In addition, it does not have the classical security loopholes, e.g. deceived by an intercept-resend attack or a correlated jamming attack. An experimental demonstration of attack detection using the technique was presented for an ideal fibre tapping attack that taps 1% of the ongoing light in a 10 dB loss channel. Detection takes 0.424 s for our 12.5 MHz repetition rate system. In addition the technique was used to detect an ideal correlated jamming attack in the channel. Hence, although the ideal correlated jamming attack maintains the light power, the excess noise of the received quantum modulated signal increased by 0.5 shot noise units within 0.04 s. Advanced statistical methods, i.e., CUSUM, have been used to increase the sensitivity by accumulating the tiny changes caused by an ideal eavesdropper. In practice, we can further increase the speed by increasing the repetition rate of the quantum detection system.

The quantum alarm is simpler than CV-QKD systems and can operate at longer ranges with a monitoring accuracy of better than 1 system shot noise (snu) at 100 skm. But it is not designed to produce cryptographic keys. It enables sensitive intruder detection but is not intended to encrypt data transmission. In practice, its precision can be further increased by utilizing a better detection system, employing a longer estimation block length, or cooperating with classical encryption methods.

Furthermore, the quantum monitoring system monitors suspicious changes in the quantum signal with the help of advanced data processing algorithms, e.g. CUSUM. Hence, unlike the CV-QKD system which is very sensitive to channel excess noise and receiver system noise^57^, the quantum monitoring is potentially more compatible with current optical infrastructure, as it lowers the system requirements and potentially allows optical amplifiers^46^ and higher system bandwidth. In addition, the quantum monitoring system can easily be introduced with high data-rate communication links length up to 100 s km.

## Methods

### Quantization noise caused by displacement

The quantum modulated signal is required to be displaced in amplitude to classical level to avoid being distinguished from the intensity. However as analyzed in Ref.^58,59^, the signal intensity offset would cause extra measurement noise measurement noise ${\varepsilon }_{m}$ due to the finite dynamic range $\left[-{x}_{m},{x}_{m}\right]$ of detector, given by:

$${\varepsilon }_{m}=\frac{1}{{N}_{0}\sqrt{2\pi {V}_{B}}}{\int }_{-\infty }^{-{x}_{m}}{(x+{x}_{m})}^{2}{e}^{-\frac{{(x-\alpha {^{\prime}})}^{2}}{2{V}_{B}}}dx +\frac{1}{{N}_{0}\sqrt{2\pi {V}_{B}}}{\int }_{-\infty }^{-{x}_{m}}{(x-{x}_{m})}^{2}{e}^{-\frac{{(x-\alpha {^{\prime}})}^{2}}{2{V}_{B}}}dx$$

The same problem exists for our detection system which consists of an Analog to Digital Converter (ADC) whose quantization noise is directly proportional to the measurement range. The quantization noise is given by^47^

$${\varepsilon }_{q}=\frac{1}{{N}_{0}}\left[0.5\times \frac{{x}_{m}-(-{x}_{m})}{{2}^{M}}\right]$$

where M is the number of bits of the ADC. In our proof of principle demonstration experiment, due to the use of classical coherent communication detector, the dynamic range is much larger than quantum detectors with an input power limit of 5 mw. However, the quantization noise cannot be neglected.

To measure the quantization noise at different measurement range, we continuously measured the same shot noise with the same LO power. As we have calibrated the shot noise and electronic noise in the previous measurements, the increase in the measured noise for the scale is considered as from the quantization noise. By subtracting the baseline noise, we can see the influence of the quantization noise on the shot noise and electronic measurement and derive the maximum input power we can have while having an accurate measurement.

**Figure 8:**
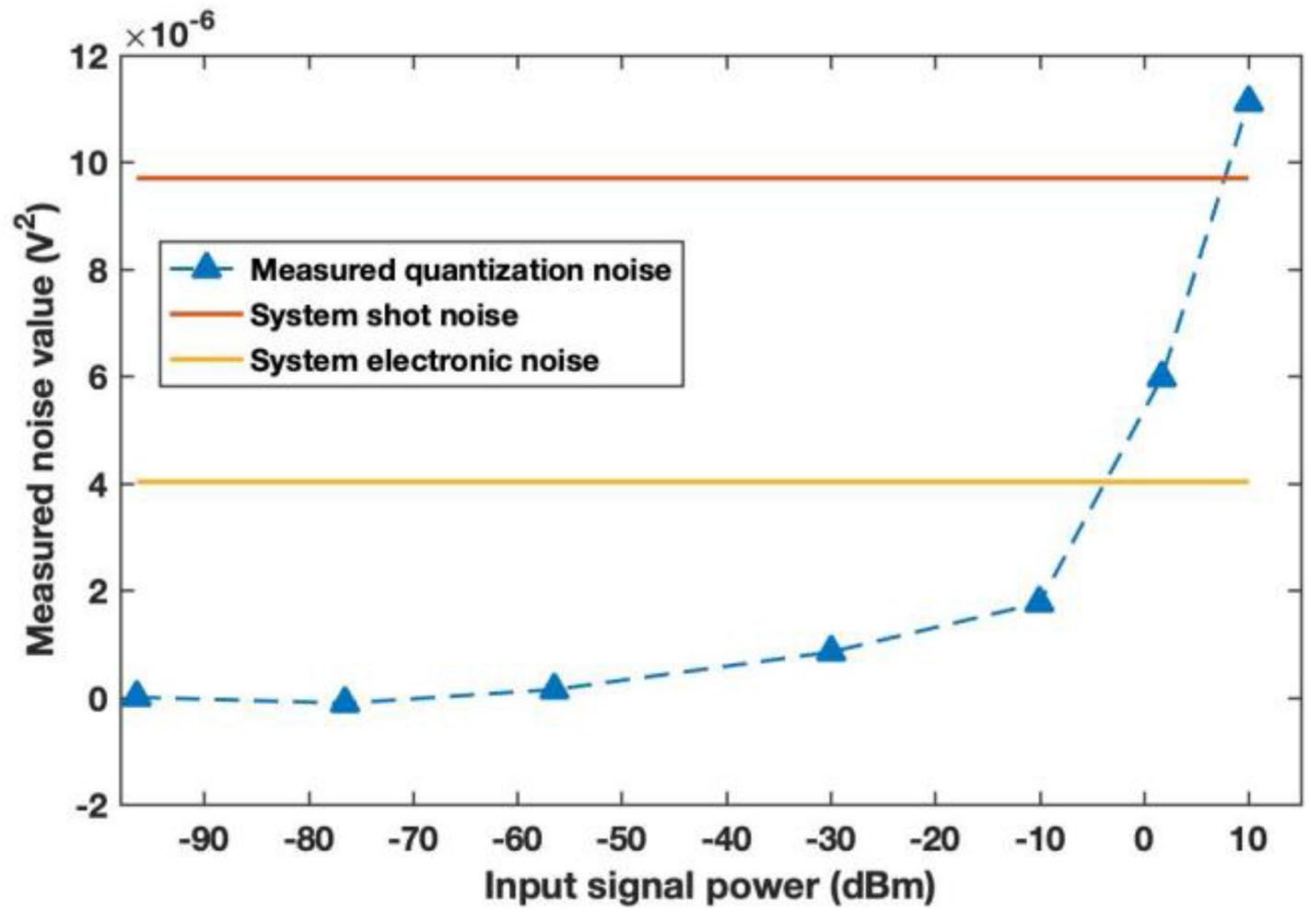
Measured quantization noise caused by different input optical signal power. The simulation is calculated for a detection system of efficiency 0.4, detector electronic noise of 0.5 shot noise, signal wavelength at 1550 nm, signal pulse duty cycle of 0.25, and ADC card bit number M of 10 or 16.

The results are shown in Fig. 8. As can be seen from the figure, the quantization noise remains at a negligible level when the resolution is less than − 30 dBm. However, if we want to increase the displacement further, the quantization noise will increase considerably and exceeds the system electronic noise.

### The CUSUM algorithm

As can be seen from algorithm 1, we calculate the cumulative sum ${S}^{i,d}\left[k\right]$ and the decision function ${G}_{\Upsilon }^{i,d}[k]$ recursively at every round of detection. By applying the algorithm, we can thus monitor the small changes in both quantum channel loss T and also the excess noise $\xi$ precisely. The change point ${n}_{c}$ will be returned when the decision function exceeds the pre-set threshold. The most likely change magnitude $\widehat{\delta }$ can be set with respect to a reasonable value that Eve would be able to decode the classical message or copy the quantum alarm signal, e.g. 0.1 dB for a fiber tapping attack or 2 snu for an intercept-and-resend attack. The detection threshold could be set according to the average run length function (ARL). For instance, we can measure ARL of the false alarm and the detection delay. These two parameters are dependent on the threshold $h$ directly and we can thus adjust the value for a desired security condition. More information about the CUSUM algorithm and approaches to evaluate the ARL function can be found in Refs.^60,61^.

**Figure:**
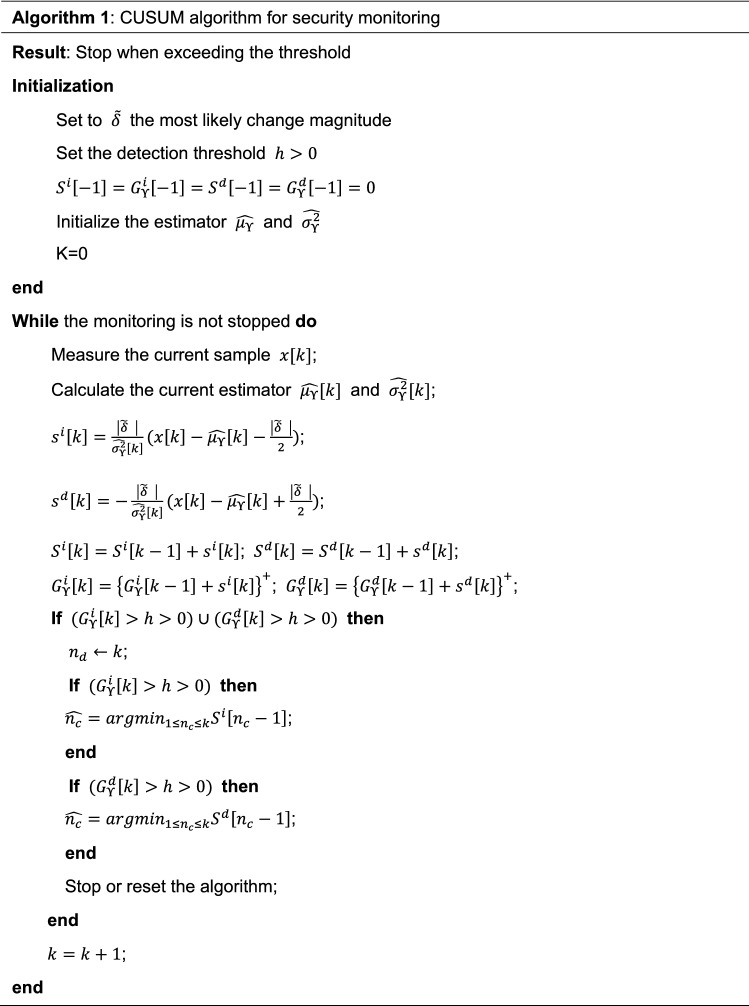
Algorithm 1: Suboptimal two-sided CUSUM algorithm for security monitoring post-processing.

### Experimental set-up details

**Figure 9:**
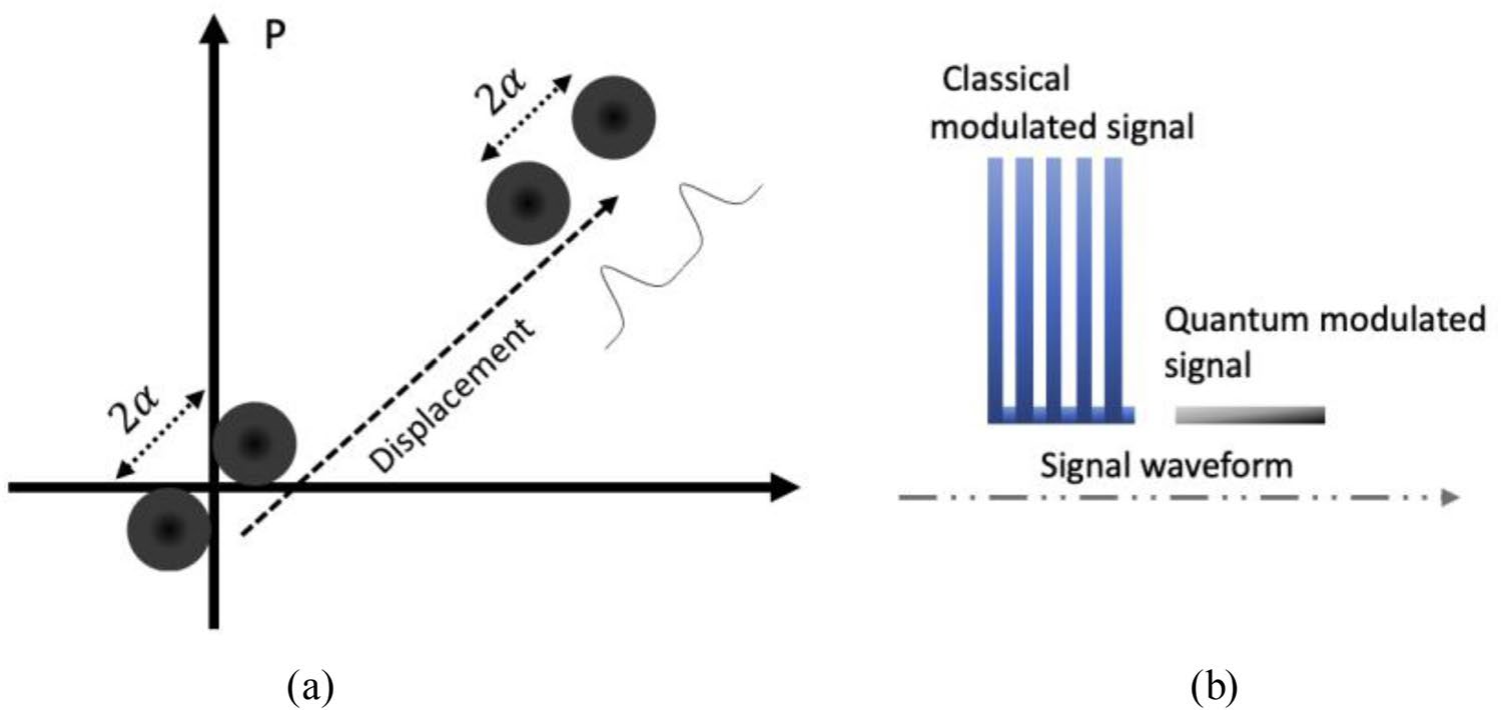
(**a**) Phase space illustration of the quantum modulated signal. (**b**) Signal waveform pattern.

As introduced in “Results”, we switch between the classical modulated and quantum modulated signal. Specifically, in our principal demonstration system, at Alice’s side, we generate both LO and signal pulses from the same continuous-wave laser at 1550 nm. The pulse width is 10 ns long with the repetition rate of 25 MHz. For our classical modulated signal, we employ the simplest classical ASK modulation scheme, i.e. the on–off keying, with the data rate of 1 Gb/s. For quantum signal, we choose the two-state modulation scheme which can be realized using single amplitude modulator to produce the displaced signal. The phase space illustration of our two quantum states is illustrated in Fig. 9a. For simplicity, we send the quantum modulated and classical signal in a deterministic order and the data patterns are illustrated in Fig. 9b. As a result, the classical signals are sent at a higher bandwidth with 10 bits encoded on a single optical pulse. In order to remove the influence of the input laser power fluctuations, we monitor the input power by splitting 90% of the light to a photodiode which continuously measures and records the power fluctuation. Regarding the LO pulse, it is sent via a separate path which is usually used in the initial experiments of LLO CVQKD system. We engineered the LO path to be the same length as the signal path by adding variable fiber delay based on a Faraday mirror. All the components are polarization maintaining to ensure a stable detection of the quantum signal.

**Figure 10:**
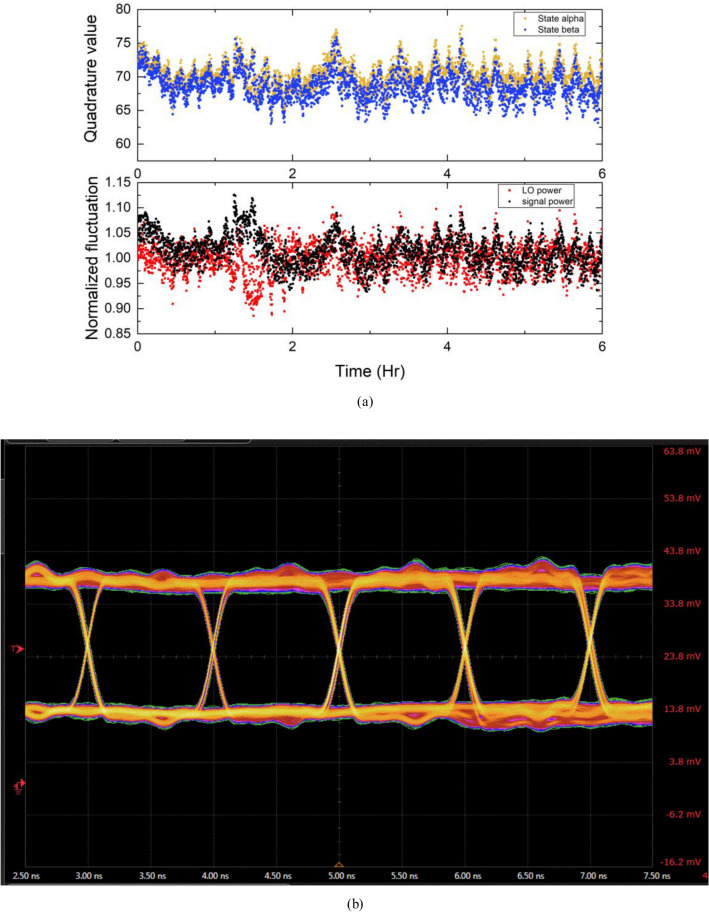
(**a**) Example of received quantum modulated signal and the recorded LO and signal fluctuation over 6 h. (**b**) The received eye diagram of classical OOK signal.

At the receiver, we utilized a low-cost heterodyne receiver which was designed for classical coherent communication to detect the quantum modulated signal, whose input power limit is 5 mW. So that, there is no question raised by the dynamic range of the detector. We measure both the X and P quadratures of the received signal. As shown in the phase space diagram of the two-state modulation, the quantum signal can be seen as unidimensional, and we can easily recover the quantum modulation from the amplitude. The received LO power is about 300 μW which leads to a LO photo number of 10^8^ photons per pulse. An example of the received quantum signal along with the record LO and signal input power fluctuation is illustrated in Fig. 10a. The eye diagram of received classical OOK signal is also presented in Fig. 10b.

## Data Availability

Additional data related to this publication is available at https://doi.org/10.17863/CAM.77727.
